# Psychological comorbidities in epilepsy: a cross-sectional survey among Ghanaian epilepsy patients

**DOI:** 10.4314/gmj.v55i2.5

**Published:** 2021-06

**Authors:** Patrick Adjei, Kodwo Nkromah, Albert Akpalu, Sammy Ohene, Peter Puplampu, Elvis T Aboagye, Vincent Ganu, Stella Nartey, Kenneth Ae-Ngibise

**Affiliations:** 1 Department of Medicine, Korle Bu Teaching Hospital, Accra, Ghana; 2 Department of Medicine and Therapeutics, University of Ghana Medical School, College of Health Sciences, University of Ghana, Accra, Ghana; 3 Department of Psychiatry, University of Ghana Medical School, College of Health Sciences, University of Ghana, Accra, Ghana; 4 Kintampo Health Research Centre, Post Office Box 200, Kintampo, Bono East Region, Ghana

**Keywords:** Epilepsy, affective disorders, anxiety, prevalence, Brief Symptom Inventory

## Abstract

**Objective:**

To evaluate the prevalence and patterns of psychiatric disorders in epilepsy patients at the Korle-Bu Teaching hospital, Accra, Ghana.

**Design:**

The study design was a cross-sectional survey

**Setting:**

The study was conducted at the Neurology Clinic of the Department of Medicine and Therapeutics, Korle-Bu Teaching hospital, Accra, Ghana.

**Participants:**

A total of one hundred and sixty-six patients diagnosed with epilepsy aged at least 18 years and accessing services at the neurology clinic participated in the study.

**Main Outcome Measure:**

Prevalence and patterns of psychiatric disorders among patients diagnosed with epilepsy using the Brief Symptom Inventory.

**Results:**

The mean age for onset of epilepsy was 20.1 ± 16.9 years, and generalized epilepsy (73.2%) was the major type of epilepsy identified. The aetiology of the epilepsy condition was unknown in most patients (71.1%). The estimated mean Brief Symptom Inventory scores in all the nine diagnostic psychiatry characteristics (Depression, Anxiety, Somatization, Hostility, Phobic Anxiety, Obsessive Compulsive Disorder, Psychoticism, Interpersonal Sensitivity, and Paranoid Ideation) were higher in the epilepsy patients compared to the normative data scores for non-patients. Global Severity Index scores for females were significantly higher (p=0.002) than the scores for males on all the psychological outcomes except hostility.

**Conclusion:**

Psychological disorders were prevalent among epilepsy patients, with females more likely to experience psychological problems than males. The findings call for a holistic approach in managing epilepsy to highlight and manage some exceptional psychological comorbidities.

**Funding:**

None declared

## Introduction

There are increasing reports of psychiatric comorbidities (depression, anxiety, somatization, hostility, phobic anxiety, obsessive-compulsive disorder, psychoticism, interpersonal sensitivity, and paranoid ideation) in patients who have epilepsy.^1^,^2^ Despite the huge challenge to healthcare delivery across populations, the clinical presentation and aetiology of the disease are yet fully comprehended. Epilepsy stands out in neurologic diseases due to the myths and beliefs linked to the condition in different cultures.^3^ The relationship between epilepsy, seizures and emotional disorders has not been consistent among neurologists and researchers.^4^ Psychiatric disorders often affect 32% - 41% of persons with epilepsy.^4^,^5^ There are reports of higher incidence of cognitive, behavioural and psychiatry symptoms in epilepsy than in the general population.^6^,^7^ Common psychiatric comorbidities found in epilepsy patients include depression, neuroses (non-psychotic anxiety disorders), risk of suicidal thought and psychosis, which affects their quality of life.^8^,^9^

The associated morbidity and mortality coupled with poor quality of life characterise epilepsy accounts for 10% of worldwide neurologic disorder burden.^10^

Medical conditions like hypoglycemia, sleep disorders, migraines, transient ischemia attacks, paroxysmal movement disorders and transient global amnesia on some occasions are also misdiagnosed for epilepsy.^11^ Most clinicians miss the clinical diagnosis of epilepsy due to the uncommon characteristic presentation of the disease.^12^ The clinical presentations of epilepsy with pockets of gradual or spontaneous psychiatry features during seizures may have a shared pathophysiological underlying mechanism.^13^ However, this remains to be elucidated. Even though most individuals with epilepsy may live normal emotional and stable minded life, psycho-behavioural symptoms manifest in most patients.^14^ Epilepsy management focus on the disease, whilst psychiatry associated comorbidities are overlooked in patients. Psychiatry disorders in epilepsy go unrecognized and untreated due to limited time, inadequate training, and clinicians' averseness to refer patients for psychiatry symptoms in most resource-limited settings.^12^ The prevalence of psychiatry comorbidities in epilepsy may complicate diagnosis and treatment, worsen prognosis, and increase health services cost, posing a huge socio-economic burden, culminating from the long-term ill-health, over dependence and mortality.^15^

Current epidemiological findings suggest a bidirectional correlation between epilepsy and mood disorders, with a prevalence of 20 - 30%.^16^ The effective treatment and management of epilepsy therefore necessitates the accurate identification and diagnosis of associated psychiatry symptoms. However, data on psychiatry features manifestation in epilepsy patients in the sub-region are scanty and limited to depression and anxiety. This study had highlighted and explored the psychiatry features manifestation in epilepsy patients relative to the general population, generating data to better understand the underlying pathophysiology. We evaluated the prevalence and patterns of affective and anxiety disorders in persons with epilepsy.

## Methods

### Study design and Participants

This was a cross-sectional study involving epilepsy patients on routine visits for treatment and management at the neurology clinic in the department of medicine, Korle Bu Teaching Hospital (KBTH), Accra, from September to December 2016. The hospital serves as the leading national referral point of care for the Southern part of Ghana as well as some neighboring West African countries.

We identified patients aged 18 years and above, clinically diagnosed to have epilepsy prior to or at the start of accessing services at the neurology clinic and recruited them consecutively by convenient sampling after signed consent. Diagnosed epilepsy patients with evidence of any diagnosed psychiatry disorder, age below 18 years, critically ill patients and those on admission were exempted from the study.

### Data collection and tools

The Brief Symptom Inventory (BSI) qualitative tool, a 53-item self-report questionnaire was used to obtain responses from study participants. The BSI tool has nine subscales designed to assess individual symptoms grouped: somatization, phobic anxiety, obsession-compulsion, depression, hostility, anxiety, paranoid ideation, interpersonal sensitivity and psychoticism. The tool has high internal consistency ranging between 0.71 – 0.98 from one dimension to the other^17^ and has been previously validated and used in the Ghanaian population by Oti-Boadi and colleagues.^18^ Cronbach's alpha reliability coefficient of 0.97 was obtained from the participants' response. Averagely participants spent 12 minutes in responding to the questions in the BSI tool. The BSI assesses psychological distress in terms of the nine primary symptom subscales stated earlier and three summary scores called global indices. In the nine subscales, psychological distress was assessed using a 5 - point Likert scale scoring from 0 (not at all) to 4 (extremely true) over the preceding seven days. The global indices measure current or past level of symptomatology, intensity of symptoms, and number of reported symptoms.^17^ The three global indices of distress are Global Severity Index (GSI), Positive Symptom Distress Index (PSDI) and Positive Symptom Total (PST). However, only GSI was calculated and used in the current analysis. Participants' socio-demographic characteristics (age, sex, ethnicity, religion, educational status, marital status and employment status) and clinical data (age of epilepsy onset, epilepsy type, etiology, number of seizures in the last 12 months, anti-epilepsy drugs taken, adherence to medication, family history of disease and stigma perception) were obtained by clinical psychologist using an additional interviewer-administered questionnaire.

### Data analysis

Data were analyzed with the Statistical Package for Social Sciences (SPSS) version 20. The mean score of dependent variables (psychiatry comorbidities assessed with the BSI instrument) for individual participants were estimated from the raw score. These estimated mean scores were then compared to the normative data mean values and analyzed. The BSI administration and response scoring, procedure manual^19^ includes normative data for four different samples: non-psychiatric adults, adolescent aged 13-17, adult psychiatric outpatients and adult psychiatric in-patients. These normative data list BSI mean raw scores for the 9 dimensions and 3 global scores for each of the four samples^19^ were used for the comparative analysis. Measured independent variables in the study (age, type of anti-epilepsy drug, number of anti-epilepsy drugs taken, age of disease onset, educational level, employment status, marital status, and seizure frequency) were presented as proportions and frequencies. Analysis of variance was used to determine the difference between the individual psychiatry comorbidities mean score of participants and the normative data mean score after satisfying the normality, equal variance and independence of data assumptions.

### Ethical considerations

The study protocol was reviewed and approved by the Ethical and Protocol Review Committee, Korle Bu Teaching Hospital, KBTH-IRB 0008/2016. Informed consent was obtained from all study participants.

## Results

A total of 200 eligible patients were selected for the cross-sectional survey. Thirty-four (17.0%) did not complete more than 7 questions in the BSI instrument, hence the remaining 166 patients with fully completed responses were included in all analysis. Table 1 shows the distribution of demographic characteristics of participants in the study.

**Table 1:** Distribution of demographic characteristics of study participants

Characteristic (N=166)	Number (%)
**Diagnosis**	
**Generalized Epilepsy**	122 (73.5)
**Focal Epilepsy**	44 (26.5)
**Treatment Type**	
**Mono**	94 (56.6)
**>1**	72 (43.4)
**Sex**	
**Female**	88 (53.0)
**Male**	78 (47.0)
**Marital Status**	
**Single**	113 (68.1)
**Married**	50 (30.1)
**Widowed/Divorced**	3 (1.8)
**Education**	
**None**	10 (6.0)
**Primary/JHS**	28 (16.9)
**SHS^+^**	60 (36.1)
**Intermediary**	21 (12.7)
**Tertiary**	47 (28.3)
**Ethnicity**	
**Akan**	71 (42.8)
**Ewe**	26 (15.7)
**Ga-Adangbe**	51 (30.7)
**Mole-Dagbani**	10 (6.0)
**Guan**	8 (4.8)
**Religion**	
**Christianity**	150 (90.4)
**Islam**	15 (9.0)
**Traditional/Other**	1 (0.6)
**Employment**	
**Employed**	78 (47.0)
**Unemployed**	40 (24.1)
**Students**	48 (28.9)

**Figure 2:**
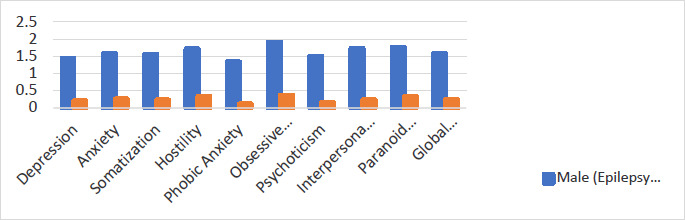
Comparison of mean scores of male epilepsy patients with normative BSI scores of adult male non epilepsy patients for all nine subscales and GSI

**Figure 3:**
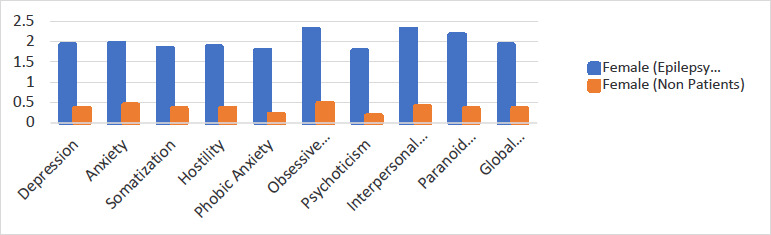
Comparison of mean of female epilepsy patients with normative BSI scores of adult female non epilepsy patients for all nine subscales and GSI

There was a significant difference between epilepsy patients' response mean score and normative data, with epilepsy patients scoring higher in all nine psychological outcomes Table 2. Independent analysis of the BSI response scores on sex showed high mean BSI scores in the female patients with epilepsy compared to normative BSI scores for patients without epilepsy as seen in Figure 2 and 3, and the detailed comparison with all reference scores as shown in supplementary materials (S1-S6).

**Table 2:** ANOVA results of male and female patients with epilepsy on the nine subscales of the BSI and GSI

	Males	Females	Sum of Squares	Df	Mean Square	F	P- value
**Depression**	1.44 (0.53)	1.92 (0.88)	9.137	1	9.137	16.568	.000*
			88.236	160	.551		
			97.373	161			
**Anxiety**	1.58 (0.67)	1.95 (0.95)	5.591	1	5.591	8.050	.005*
			111.810	161	.694		
			117.401	162			
**Somatization**	1.55 (0.55)	1.83 (0.77)	3.113	1	3.113	6.721	.010*
			73.654	159	.463		
			76.767	160			
**Hostility**	1.71 (0.82)	1.88 (0.83)	1.072	1	1.072	1.571	.212
			109.164	160	.682		
			110.236	161			
**Phobic Anxiety**	1.33 (0.61)	1.79 (0.97)	8.010	1	8.010	11.730	.001*
			107.894	158	.683		
			115.904	159			
**Obsessive Compulsive Disorder**	1.91 (0.76)	2.30 (0.99)	5.958	1	5.958	7.441	.007*
			127.321	159	.801		
			133.280	160			
**Psychoticism**	1.50 (0.60)	1.77 (0.93)	3.010	1	3.010	4.764	.031*
			101.734	161	.632		
			104.744	162			
**Interpersonal Sensitivity**	1.71 (0.86)	2.31 (1.21)	13.872	1	13.872	12.177	.001*
			179.989	158	1.139		
			193.861	159			
**Paranoid Ideation**	1.76 (0.74)	2.17 (1.05)	6.865	1	6.865	8.057	.005*
			136.333	160	.852		
			143.198	161			
**Global Severity Indices**	1.58 (0.49)	1.77 (0.75)	4.193	1	4.193	10.043	.002*
			60.960	146	.418		
			65.153	147			

Df - degrees of freedom, F=F-statistic

* = P < 0.05, standard deviations appear in parentheses below the means

The Global Severity Index (GSI) scores for females were significantly higher than the scores for males on all the psychological outcomes, except on hostility depicted in Table 2.

## Discussion

There are increasing reports of psychiatric comorbidities (depression, anxiety, somatization, hostility, phobic anxiety, obsessive compulsive disorder, psychoticism, interpersonal sensitivity, and paranoid ideation) in epilepsy patients.^2^ Reported prevalence of these disorders in epilepsy has not been consistent in the sub-region.^20^,^21^,^22^ In this cross-sectional descriptive study, we assessed the prevalence of specific psychiatry comorbidities in epilepsy patients. The estimated mean BSI scores obtained from epilepsy patients in the study population were higher in all the nine assessed psychiatry characteristics compared to the normative data in a reference population. The observed difference between the scores of the epilepsy patients and the comparative non-patient groups were statistically significant in all features.

This finding is consistent with earlier studies that observed high frequencies of psychiatric disorders among epilepsy patients compared to the general population.^21^,^23^ The results is also similar to the multi-country study conducted in the sub-region that recorded psycho-social and medical comorbidities to be significantly prevalent among epilepsy patients relative to community controls.^24^ Increased psychiatry comorbidities reported in studies in neighboring Nigeria among epilepsy patients.^25^,^26^ A study conducted at tertiary centers also identified 40-60%^27^ prevalence of psychiatric features in epilepsy patients, compared to 20% prevalence in a population based study.^28^ This variation in the prevalence of psychiatry features in epilepsy could be attributed to the types of patient studied, the type of psychiatry disorder, duration of the study, and the specific instrument used for assessment.^26^

Depression, non-psychotic anxiety and psychoses have been reported as the most prevalent psychiatric features in epilepsy patients.^23^,^29^ Depression and anxiety particularly share some biological and pathophysiological mechanisms that are linked to limbic system dysfunction with the phenomenon yet to be fully confirmed. About 25-40% of persons with epilepsy develop marked depression and psychotic symptoms in the course of illness.^30^ Alterations in cognitive activity from seizures could account for the depression, and the psychosocial stress associated with this highly stigmatized disorder may worsen the anxiety and other psychiatry disorders.^29^ Due to the general medical condition, mood and other psychotic manifestations in epilepsy are usually anticipated.

The correlation between psychiatric comorbidities and epilepsy has been explained with three thoughts; those disorders may share some common neurological pathogenesis with epilepsy, inducing the observed psychiatric disorders.^31^ Epilepsy and psychiatric disorders could also have psychosocial stress relationship.^32^ The bidirectional relationship between epilepsy and depression confirmed in different populations could support the likely potential of overlapping mechanism in manifestation. Lastly, convulsive seizure is thought to influence induction ischemia and inflammation in the brain, culminating in subtle brain damage and maybe, the accompanying psychiatric disorders.^33^,^34^,^35^

Several reasons have been linked to the observed psychological distress in patients with epilepsy. Society's lack of understanding of epilepsy makes it a strongly felt psychosocial burden and affected individuals may try to conceal the condition. Feelings of anger, frustration, embarrassment, and vulnerability may develop as a result of society's attitude towards the illness.^36^ In addition, poor adherence to anti-epilepsy drugs (AEDs) might affect patient's seizure control, cognitive impairment, and behavioral disorders commonly observed in epilepsy patients.^37^ However, medication non-adherence had no influence in the observed psychiatry features in the current study since the level of medication compliance was high in the study population. Cognitive, behavioral and motor impairment comorbidities of epilepsy in the sub-region have not been fully described in previous studies and may account for the lack of social functioning and acceptance manifestation.^38^ Emotional disturbances that characterizes epilepsy are thought to be caused by the interplay of adverse effects of cerebral function resulting from prolonged and repeated seizures or anticonvulsant drug toxicity and social functioning.^23^ Societal attitudes that describes epilepsy as a shameful condition coupled with the unjust fear in patients as a result of perceived stigma may influence the associated cognitive comorbidities.^39^

Females had significantly higher psychiatric characteristic scores on the assessed features compared to males in the study except hostility. This finding is in line with a previous report that observed high psychiatry comorbidities in females than their male counterparts.^36^ Previous studies had shown that, epilepsy is more prevalent in females compared to males.^25^,^26^ The 46.7% (males) and 53.3% (females) in the current study is comparable to 50.8% (males) and 49.2% (females) reported in Kumasi,^21^ 49.1% (males) and 50.9% (females) in Kintampo,^40^ and 48% (males) and 52% (females) in Zambia,^22^ and 50.3% (males) and 49.7% (females) in India^31^ respectively.

Generalized epilepsy constituted the greater proportion of epilepsy type, with only 26.3% having partial epilepsy in our cohort. Focal epilepsy has been associated with poor neurobehavioral outcomes in Africa with increased manifestation of cognitive impairment and neurologic disorders.^24^ The difference in study design (retrospective against cross-sectional prospective recruitment in the current study) could account for the difference. However, possible misclassification of the seizure types stated in the previous study as limitation due to the reported lack of electroencephalogram (EEG) and neuroimaging that affected the classification of patients with focal-onset seizures with secondary generalization into the generalized group may have been a factor.^21^

Despite the above findings, the study had limitation due to its cross-sectional study design which limited the level of assessment of patients. The sample size for the study was small and therefore limits the generalizability of the study findings. In addition, we recognized these are symptoms that does not translate directly to a diagnosis of the psychological comorbidity. Nevertheless, the study highlighted significant findings of psychological distress among patients with epilepsy. Intervention programs may be put in place to help identify components of these features for treatment which in the long run will improve treatment and quality of life of patients with epilepsy.

## Conclusion

The current findings highlight some critical challenges faced by epilepsy patients in their daily lives; struggles with depression, anxiety, somatization, and other psychological distress. These distresses may be borne out of social factors including stigma and negative attitudes in the society. Future studies could focus on the qualitative aspects of this study among health service providers and patients to highlight the exceptional features of psychological problems in epilepsy management.

## Figures and Tables

**Figure 1:**
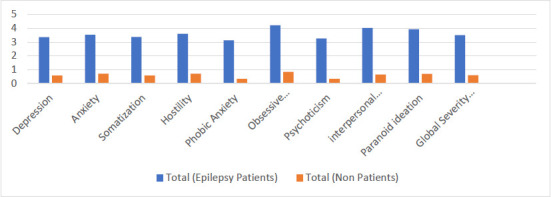
Comparison of mean scores of epilepsy patients with normative BSI scores of adult patients for all nine subscales and GSI
